# Deacetylase activity-independent transcriptional activation by HDAC2 during TPA-induced HL-60 cell differentiation

**DOI:** 10.1371/journal.pone.0202935

**Published:** 2018-08-24

**Authors:** Hyeonsoo Jung, Ji-Young Kim, Kee-Beom Kim, Yun-Cheol Chae, Yoonsoo Hahn, Jung-Woong Kim, Sang-Beom Seo

**Affiliations:** Department of Life Science, College of Natural Sciences, Chung-Ang University, Seoul, Korea; Hirosaki University Graduate School of Medicine, JAPAN

## Abstract

The human myeloid leukemia cell line HL-60 differentiate into monocytes following treatment with 12-*O*-tetradecanoylphorbol-13-acetate (TPA). However, the mechanism underlying the differentiation of these cells in response to TPA has not been fully elucidated. In this study, we performed ChIP-seq profiling of RNA Pol II, HDAC2, Acetyl H3 (AcH3), and H3K27me3 and analyzed differential chromatin state changes during TPA-induced differentiation of HL-60 cells. We focused on atypically active genes, which showed enhanced H3 acetylation despite increased HDAC2 recruitment. We found that HDAC2 positively regulates the expression of these genes in a histone deacetylase activity-independent manner. HDAC2 interacted with and recruited paired box 5 (PAX5) to the promoters of the target genes and regulated HL-60 cell differentiation by PAX5-mediated gene activation. Taken together, these data elucidated the specific-chromatin status during HL-60 cell differentiation following TPA exposure and suggested that HDAC2 can activate transcription of certain genes through interactions with PAX5 in a deacetylase activity-independent pathway.

## Introduction

Acute myeloid leukemia (AML) is a type of cancer that occurs in the bone marrow, blood, and other tissues of the hematopoietic system. AML is now curable in 35 to 40% of adult patients under 60 years of age and in 5 to 15% of patients over the 60 years old [1]. However, the average age of AML patients is approximately 68 years, and most of these patients cannot be treated with intensive chemotherapy because of the unexpected side effects and toxicity of the chemotherapeutic agents [2]. For this reason, understanding the mechanisms underlying the action of these agents is indispensable for their application to patients.

HL-60 cells, a human AML cell line, are great models to study the properties of leukemia cells and to test chemotherapeutic agents. They have the ability to differentiate into granulocytes, monocytes, macrophage, and eosinophils following induction by various chemicals [3]. For instance, all-*trans* retinoic acid (ATRA) and 12-*O*-tetradecanoylphorbol-13-acetate (TPA), which induced HL-60 cell differentiation into granulocytes and macrophages, respectively, were used for leukemia therapies [4, 5]. Although many researchers have tried to elucidate the mechanisms underlying differentiation by these agents, these mechanisms are still not well understood [6].

Epigenetic regulations, including DNA methylation, histone modification, chromatin remodeling, and non-coding RNA-mediated targeting, are crucial in many biological processes related to induction and maintenance of various cancers, including AML [7, 8]. To understand the mechanism underlying epigenetic regulation of HL-60 cell differentiation, global genomic analyses such as those on gene expression or chromatin accessibility dynamics, have been conducted [9, 10]. These studies have explained differential gene expression changes during HL-60 cell differentiation in response to various chemical agents [11–14]. To reduce the side effects and toxicity of therapeutic agents for leukemia that have global effects, direct regulation of gene expression by an epigenetic regulator has been the focus of several studies. For example, the inhibitors of epigenetic enzymes, including DNMTs, HDACs, JAK, CBP/p300, and EZH2, have been examined for possible clinical application [15–17]. Particularly, HDACs have been regarded as new therapeutic targets for leukemia [18, 19]. In addition, there have been trials to combine chemotherapeutic agents and epigenetic drugs to cure AML [17, 20, 21]. To reduce the risks of these agents, determining the mechanisms underlying the function of epigenetic regulators in HL-60 cell differentiation are important because of the global effects of these agents in leukemia cells.

Histone modifiers can function differently according to the tissue in which they are expressed or due to alternative splicing or interacting proteins. For example, the FAD-dependent amine oxidase lysine-specific demethylase 1A (LSD1) is a unique protein that catalyzes the demethylation of H3K4me2 and H3K9me2, acting as a transcriptional repressor or activator, respectively [22, 23]. Specifically, a specific LSD1 isoform activates neuronal genes and regulates neuronal differentiation [24]. In addition, the H3K27 demethylases Jmjd3 and UTX are required for general chromatin remodeling because of their catalytic activity-independent functions [25]. p300 and PCAF also have roles as co-activators that are independent from those of histone acetyltransferase [26, 27].

PAX5 (paired box 5) plays a critical role in B cell development and leukemogenesis of acute lymphoblastic leukemia [28, 29]. However, PAX5 also acts as a tumor suppressor in the B-lymphoid lineage of cells in mice [30, 31]. PAX5 can act as both a transcriptional activator and repressor, depending on specific interacting partners [32, 33].

Here, we exposed HL-60 cells to TPA and performed a chromatin immunoprecipitation followed by high-throughput deep sequencing (ChIP-seq) analysis during differentiation using acetyl-histone H3 (AcH3), RNA polymerase II (RNA Pol II), HDAC2, and H3K27me3 antibodies. From this data, we identified certain target genes that showed increased H3 acetylation, despite increased HDAC2 recruitment. We found that HDAC2 positively regulated the expression of these genes and enhanced HL-60 cell differentiation via PAX5-mediated gene activation by recruiting PAX5 to the promoters of the target genes in a histone deacetylase activity-independent manner.

## Materials and methods

### Cell culture

HL-60 cells were grown in RPMI-1640 medium, and HEK293T cells were grown in Dulbecco’s modified Eagle’s medium (DMEM) containing 10% heat-inactivated fetal bovine serum and 0.05% penicillin-streptomycin. Both cell lines were maintained at 37°C in a 5% CO_2_ atmosphere. For differentiation, HL-60 cells were seeded in a 100-mm plate at a concentration of 1 × 10^6^ per mL and treated with 32 nM TPA (Sigma Aldrich) or DMSO (Duchefa).

### Plasmid constructs

For the luciferase assay, the *IL10RA* and *RGCC* promoter regions (−998 to −1 and −1468 to +5, respectively) were amplified from genomic DNA using the primer pairs shown in S1 Table and inserted into the pGL3.0-basic vector (Promega). pcDNA3-PAX5 was subcloned into the pCMV-Flag vector using primer pairs shown in S1 Table. Short hairpin RNAs (shRNAs) against *HDAC2* and *PAX5* were designed using siRNA sequence design software (Clontech). Double-stranded oligonucleotides for shRNA plasmid construction were produced using primers at the 5′ and 3′ ends (S1 Table). These oligonucleotides were inserted into the *Age*I/*EcoR*I site of the pLKO.1 TRC vector (Addgene).

### Antibodies

The antibodies used for the ChIP assays were directed against RNA Pol II (Santa Cruz [sc-899 X]), HDAC2 (Abcam [ab12169]), AcH3 (Abcam [ab47915]), PAX5 (Santa Cruz [sc-13146 X]), and H3K27me3 (Millipore [07–449]. Antibodies against PAX5 (Santa Cruz [sc-13146 X]) and Flag (Sigma [F3165]) were used in the immunoprecipitation (IP) and PAX5 (Santa Cruz [sc-1974]) and HDAC2 (Abcam [ab12169]) were used in the immunoblot assays.

### ChIP-sequencing and data analysis

Barcoded sequence libraries were generated using TruSeq ChIP Library Preparation Kits (Illumina, CA) according to the manufacturer’s protocol, beginning with 5–10 ng of ChIP DNA from HL-60 cells. Sequencing data files were aligned to the hg19 human reference genome using Bowtie (version 1.1.2) and standard parameters. Peak calling and the analysis were performed using HOMER, which is freely available at http://biowhat.ucsd.edu/homer/. Only unique sequencing tags were considered in the analysis. Results were visualized by preparing custom tracks in the UCSC browser. Peaks were identified using a false discovery rate of < 0.1%, and IgG chromatin-derived DNAs from DMSO- or TPA-treated cells were used as controls. Identified peaks were annotated to the nearest transcription start site (TSS). Heatmaps were generated using HOMER and visualized with Java TreeView. ChIP fragment depth was calculated by extending sequencing tags by their estimated ChIP fragment lengths. The ChIP-seq data were submitted to the GEO database (GSE110566).

### Reverse transcription PCR and quantitative real-time PCR (qPCR)

Total RNA was isolated from cells using Tri-RNA Reagent (Favorgen). After synthesis, the cDNA was quantified and subjected to mRNA expression analysis. The PCR primers used are presented in S1 Table. Dissociation curves were examined after each PCR run to ensure amplification of a single product of the appropriate length. The mean threshold cycle (C_T_) and standard error values were calculated from individual C_T_ values obtained from triplicate reactions per stage. The mean normalized C_T_ value was estimated as ΔC_T_ by subtracting the mean C_T_ of *GAPDH*. The ΔΔC_T_ value was calculated as the difference between the control ΔC_T_ and the value obtained for each sample. The n-fold change in gene expression, relative to the expression in a control, was calculated as 2^-ΔΔCT^.

### Lentivirus transduction

To produce virus particles, HEK293T cells were co-transfected with plasmids encoding VSV-G, NL-BH, and shRNAs against *HDAC2* and *PAX5*. Two days after transfection, the supernatants containing the viruses were collected and used to infect HL-60 cells in the presence of polybrene (8 μg/mL).

### Luciferase assay

For the luciferase assay, HEK293T cells were seeded in 48-well plates and co-transfected with the indicated expression plasmid and a pGL3.0-*IL0RA* or pGL3.0-*RGCC* reporter plasmid using polyethylenimine. After 48 hrs, the cells were harvested and subjected to a luciferase assay (Promega). β-galactosidase activity levels were used to normalize reporter luciferase activity. Data are expressed as the means of four replicates in a single assay. All results shown are representative of at least three independent experiments.

### ChIP-qPCR assay

Cells were harvested and subsequently cross-linked with 1% formaldehyde. Briefly, 1% formaldehyde was added to the medium for 10 min at room temperature, followed by the addition of 125 mM glycine for 5 min at room temperature. HL-60 cells were centrifuged, and the resulting pellets were washed once with 1× phosphate-buffered saline. The cell pellets were resuspended in sodium dodecyl sulfate (SDS) lysis buffer (1% SDS, 10 mM EDTA, 50 mM Tris-HCl [pH 8.1]). The cells were sonicated, and the lysates were subjected to IP using the indicated antibodies. The immunoprecipitates were eluted and reverse cross-linked. Subsequently, the DNA fragments were purified for PCR amplification. Following this, the DNA fragments were purified and PCR amplified for quantification using each PCR primer pair (S1 Table). The thermal cycling conditions were as follows: 3 min at 95°C, followed by 45 cycles at 95°C for 10 s, 56°C to 60°C for 10 s, and 72°C for 30 s (Bio-Rad). The mean threshold cycle (C_T_) and standard error values were calculated from individual C_T_ values from duplicate reactions in each stage.

### IP

IP was performed to investigate the relationship between HDAC2 and PAX5 during HL-60 cell differentiation. Cells were lysed in lysis buffer (20 mM Tris-HCl [pH 7.5], 150 mM NaCl, 1 mM EDTA, 1 mM EGTA, 1% Triton X-100, 1× protease inhibitor cocktail, and 1 mM PMSF) at 4°C. Proteins were immunoprecipitated with anti-Flag or anti-PAX5 antibodies overnight at 4°C. Following, protein A/G agarose beads (GenDEPOT) were added for 4 hrs with rotation at 4°C. Bound proteins were analyzed via western blotting with anti-HDAC2, anti-PAX5, and anti-Flag antibodies.

### Fluorescence-activated cell sorting (FACS) analysis

To measure the effect of enzyme activity-independent HDAC2 function on differentiation, cells were stained with CD11b PE (12-0118-42, eBioscience) with 1% BSA and 0.5% Tween 20 in PBS for 1 hr. HL-60 cells were then subjected to FACS analysis using a BD FACSAriaTM II (BD bioscience), and the data were analyzed using BD Accuri C6 plus (BD bioscience).

### Statistical analysis

Data are expressed as the means ± standard deviations for the ChIP assay or means ± standard errors of the mean for the gene expression and luciferase assays, both based on three or more independent experiments. Statistical significance (*P* < 0.05) was calculated in Microsoft Excel. Differences between groups were evaluated by a Student’s t-test or Bonferroni test, as appropriate.

## Results

### Genome-wide quantitative profiling of chromatin modifications

**Fig 1:**
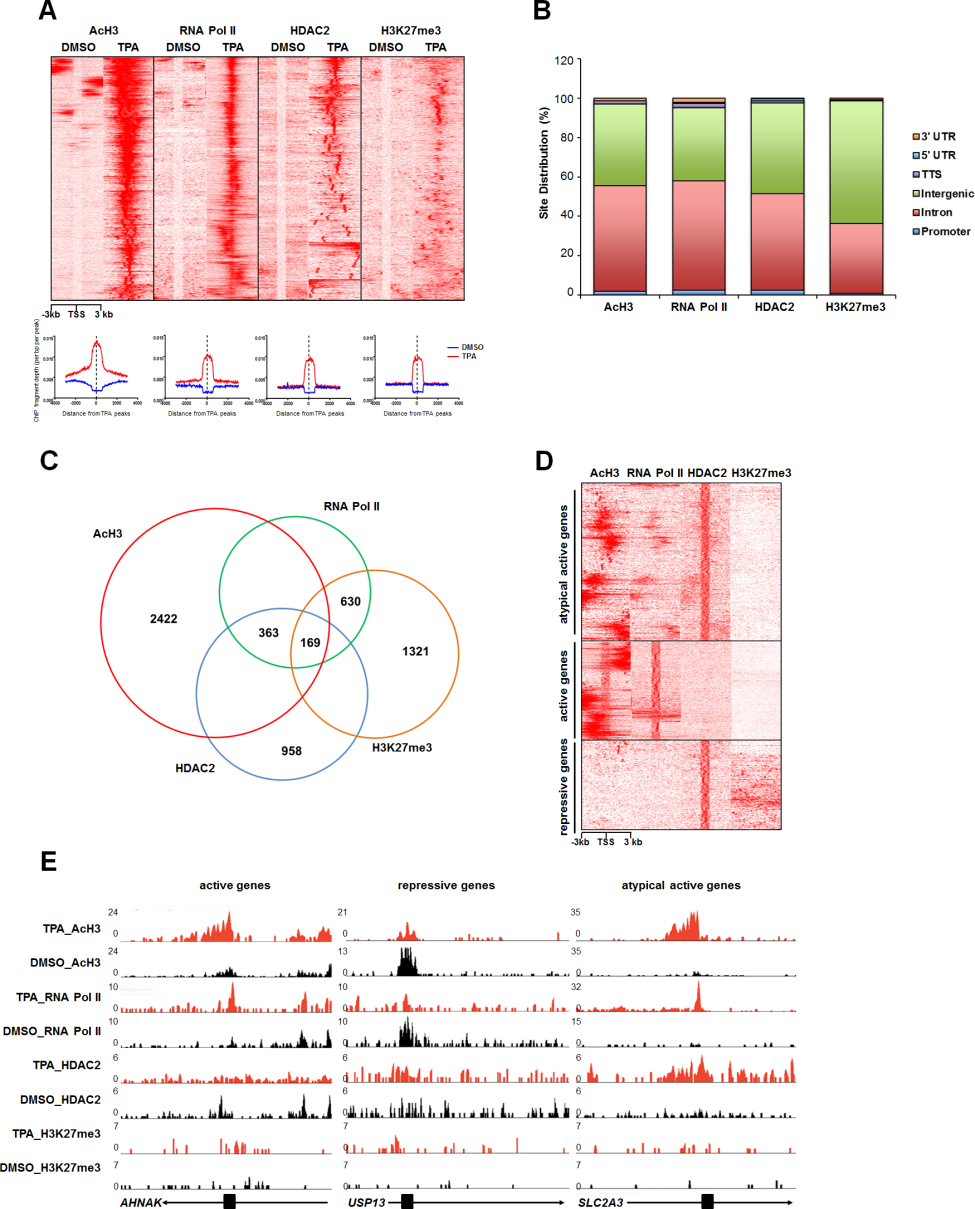
Genome-wide distribution profiles of AcH3, RNA Pol II, HDAC2, and H3K27me3 in TPA-treated HL-60 cells. (A) Heat map analysis showing AcH3, RNA Pol II, HDAC2, and H3K27me3 occupancies covering ± 3 kb region centered over each peak in DMSO- or TPA-treated HL-60 cells. The same color scales (white, no enrichment; red, high enrichment) were used for all data sets (upper panel). Merged profiles show each ChIP-seq signal around the TSS in DMSO- or TPA-treated HL-60 cells (lower panel) (B) Pie charts illustrate the genomic locations of AcH3, RNA Pol II, HDAC2, and H3K27me3 binding sites in TPA-treated HL-60 cells. (C) An area-proportional Venn diagram shows the overlap between AcH3, RNA Pol II, HDAC2, and H3K27me3 enrichment in TPA-treated HL-60 cells based on ChIP-seq data. (D) A heat map represents the binding profiles of AcH3, RNA Pol II, HDAC2, and H3K27me3 in TPA-treated HL-60 cells at the indicated target genes (± 3 kb around the TSSs). The same color scales (white, no enrichment; red, high enrichment) were used for all data sets. (E) ChIP-seq tracks of AcH3, RNA Pol II, HDAC2, and H3K27me3 in TPA-treated HL-60 cells along the *AHNAK*, *USP13*, and *SLC2A3* loci.

Recently, researchers have taken interests in epigenetic modifiers, including HDACs, as therapeutic targets and partners for existing chemotherapeutic agents [19]. However, the functions of HDACs in leukemia have not yet been fully established. Therefore, we analyzed the differential epigenetic status and gene expression regulated by HDAC2 during HL-60 cell differentiation by TPA. We determined RNA Pol II and HDAC2 occupancies and profiles of two histone modifications (AcH3 and H3K27me3) using ChIP-seq from HL-60 cells exposed to TPA or DMSO (control) for 48 hrs. Over 16 million reads were obtained for each ChIP from the sequencing run. Sequencing reads were aligned to the human reference genome, and peaks were called. We first analyzed equal numbers of reads based on ChIP-seq signal intensity from the control and TPA-treated HL-60 cells. We found that the enrichment levels of AcH3, RNA Pol II, HDAC2, and H3K27me3 were significantly increased in the TPA-treated cells compared to those in the controls (Fig 1A). We next showed the peak distribution profiles relative to those positions in nearby genes (Fig 1B). Unexpectedly, we found that most peaks of AcH3, RNA Pol II, HDAC2, and H3K27me3 occupied intron and intergenic regions of annotated genes following TPA treatment (Fig 1B). A venn diagram depicting the number of occupied sites, and the overlap of AcH3, RNA Pol II, and HDAC2 genomic binding sites in TPA-treated HL-60 cells is shown in Fig 1C. For further analysis, we compared genome-wide similarities of chromatin profiles in TPA-treated HL-60 cells, such that TPA treatment-specific peaks could be categorized into two major groups (Fig 1D). The first group was associated with typically active markers, including AcH3 and RNA Pol II or markers of repression (HDAC2 and H3K27me3). In contrast, the second group was associated with atypically active genes, which were characterized by the presence of HDAC2 as well as AcH3 and RNA Pol II. Approximately 14% of all HDAC2 peaks were in this group (Fig 1C and 1D). For example, AcH3 and RNA Pol II co-localized on the promoter regions of *SLC2A3* and other atypically active genes in HDAC2 peaks, but only AcH3 and RNA Pol II were enriched at the *AHNAK* promoter region (Fig 1E and S1 Fig). These results suggest that HDAC2 can associate with both active and repressed target genes in differentiated HL-60 cells.

### Atypical genes are activated by HDAC2 in a deacetylase activity-independent manner during HL-60 cell differentiation

**Fig 2:**
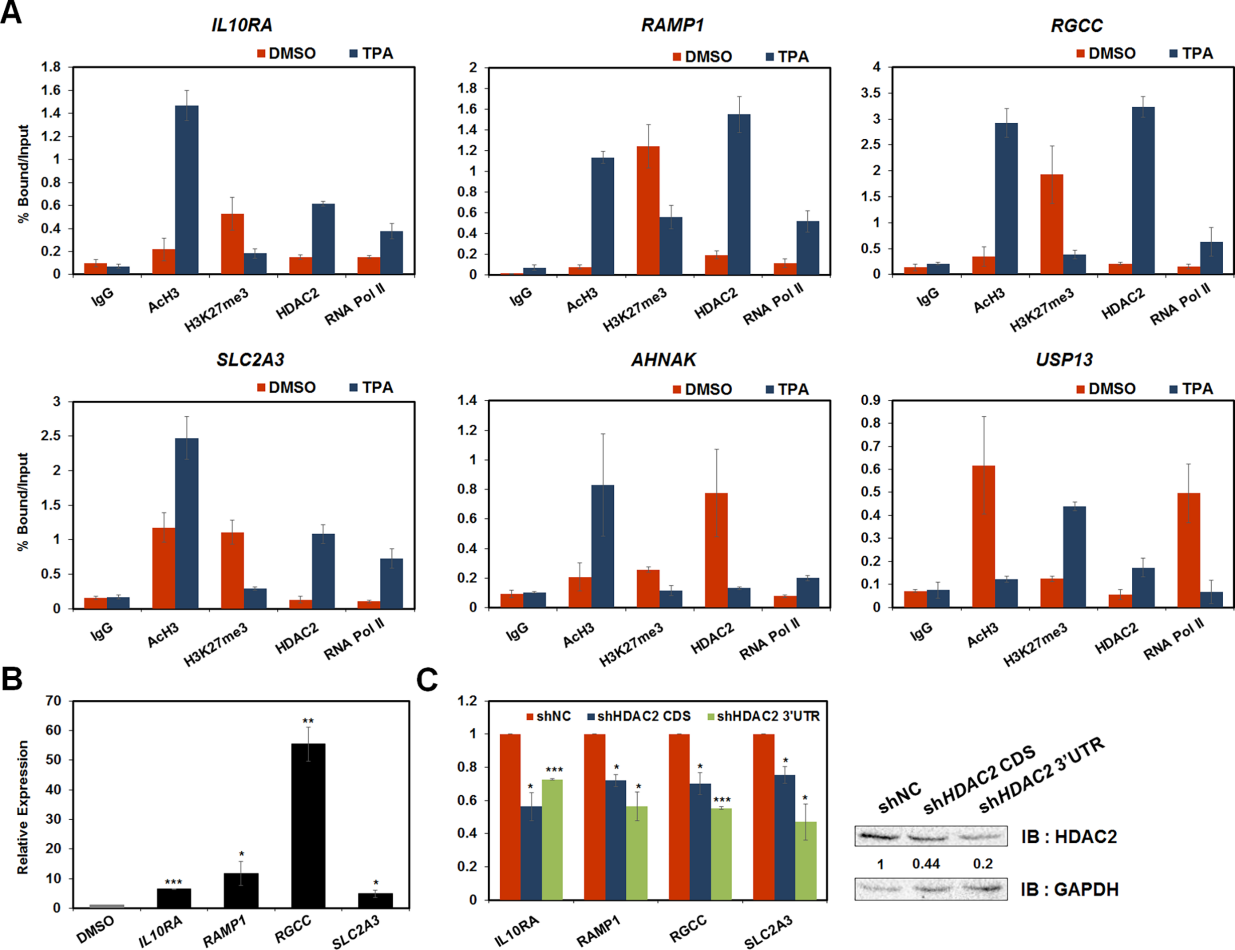
HDAC2 was recruited to the promoters of atypically active genes and induced transcription during HL-60 cell differentiation. (A–B) HL-60 cells were treated with TPA (32 nM) or DMSO for 48 hrs. (A) The occupancies of AcH3, H3K27me3, HDAC2, and RNA Pol II at the promoters of atypically active genes, typically active and repressive genes during HL-60 cell differentiation were analyzed. The data were normalized by input. These results are shown as means ± SDs (n = 3). (B) The differential changes in expression of the genes in TPA-treated HL-60 cells were confirmed by qPCR. (C) The mRNA levels of atypically active genes in *HDAC2*-depleted HL-60 cells were analyzed using qPCR. Knockdown of *HDAC2* was confirmed by western blotting. (B–C) These data were normalized by GAPDH. All results represent at least three independent experiments (± SEMs). * *P* < 0.05, ** *P* < 0.01, *** *P* < 0.001.

We categorized the genes in the ChIP-seq analysis into two groups that showed similar peaks with the typically active markers AcH3 and RNA Pol II or repressive marker H3K27me3. The recruitment of HDAC2 decreased in the first group and increased in the second group. These data showed increased histone acetylation levels and enrichment of HDAC2 in atypically active genes in HL-60 cells during differentiation in response to TPA treatment (Fig 1E). The fact that histone deacetylase recruitment resulted in increased histone acetylation may indicate that HDAC2 can regulate gene expression in leukemia in a histone deacetylase activity-independent manner. To elucidate the molecular mechanisms by which HDAC2 regulates gene expression independently of HDAC activity, we first verified the differential chromatin state changes of atypically active genes in the ChIP-seq data (Fig 2A and Panel B in S2 Fig). Differentiation of HL-60 cells was measured by the mRNA levels of the differentiation marker *CD11b* (Panel A in S2 Fig). After treatment of HL-60 cells with TPA for 48 hrs, H3 acetylation levels and RNA Pol II recruitment to the promoters of atypically active genes such as *IL10RA*, *RAMP1*, *RGCC*, and *SLC2A3*, increased, whereas H3K27me3 levels were attenuated. These differential occupancies indicate the upregulation of gene expression, even though HDAC2 recruitment also increased. In contrast, the promoters of genes typically regulated during HL-60 cell differentiation following TPA treatment, including *SGK1* and *AHNAK*, which were activated, and *MGST3* and *USP13*, which were repressed, showed differential histone marker changes indicating negative regulation of gene expression by HDAC2 (Fig 2A and Panel B in S2 Fig). Subsequently, we performed qPCR to confirm the expression of genes during HL-60 cell differentiation and found that transcription of atypically active genes and *SGK1* and *AHNAK* was enhanced, whereas *MGST3* and *USP13* expression levels were diminished (Fig 2B and Panel C in S2 Fig). To test whether HDAC2 participates in the regulation of gene expression, we also analyzed gene expression in *HDAC2*-depleted HL-60 cells. Interestingly, knockdown of *HDAC2* in HL-60 cells decreased expression of atypically active genes. These results indicated that HDAC2 positively regulates gene expression (Fig 2C).

Taken together, these data showed that HDAC2 was recruited to the promoters of atypically active genes, even though histone acetylation levels were also increased during HL-60 cell differentiation in response to TPA. These data also suggested that HDAC2 positively regulated gene expression. Because HDAC2 usually represses gene expressions by reducing histone acetylation, we hypothesized that this regulation by HDAC2 is independent of histone deacetylase activity.

### HDAC2 recruits PAX5 to the promoters of atypically active genes during HL-60 cell differentiation

To explain the aberrant HDAC2 recruitment at atypically active genes, we speculated that HDAC2 may recruit transcription factors that positively regulate gene expression. To test this hypothesis, we screened transcription factors that were bound to promoters of atypically active genes by searching for conserved DNA sequence motifs using the cisRED prediction program (http://www.cisred.org/human9). The results showed that PAX5 was the most abundant among atypically active genes, including *IL10RA*, *RAMP1*, and *RGCC*. Therefore, we decided to test whether PAX5 was a transcription factor associated with HDAC2 at atypically active genes during HL-60 cell differentiation.

**Fig 3:**
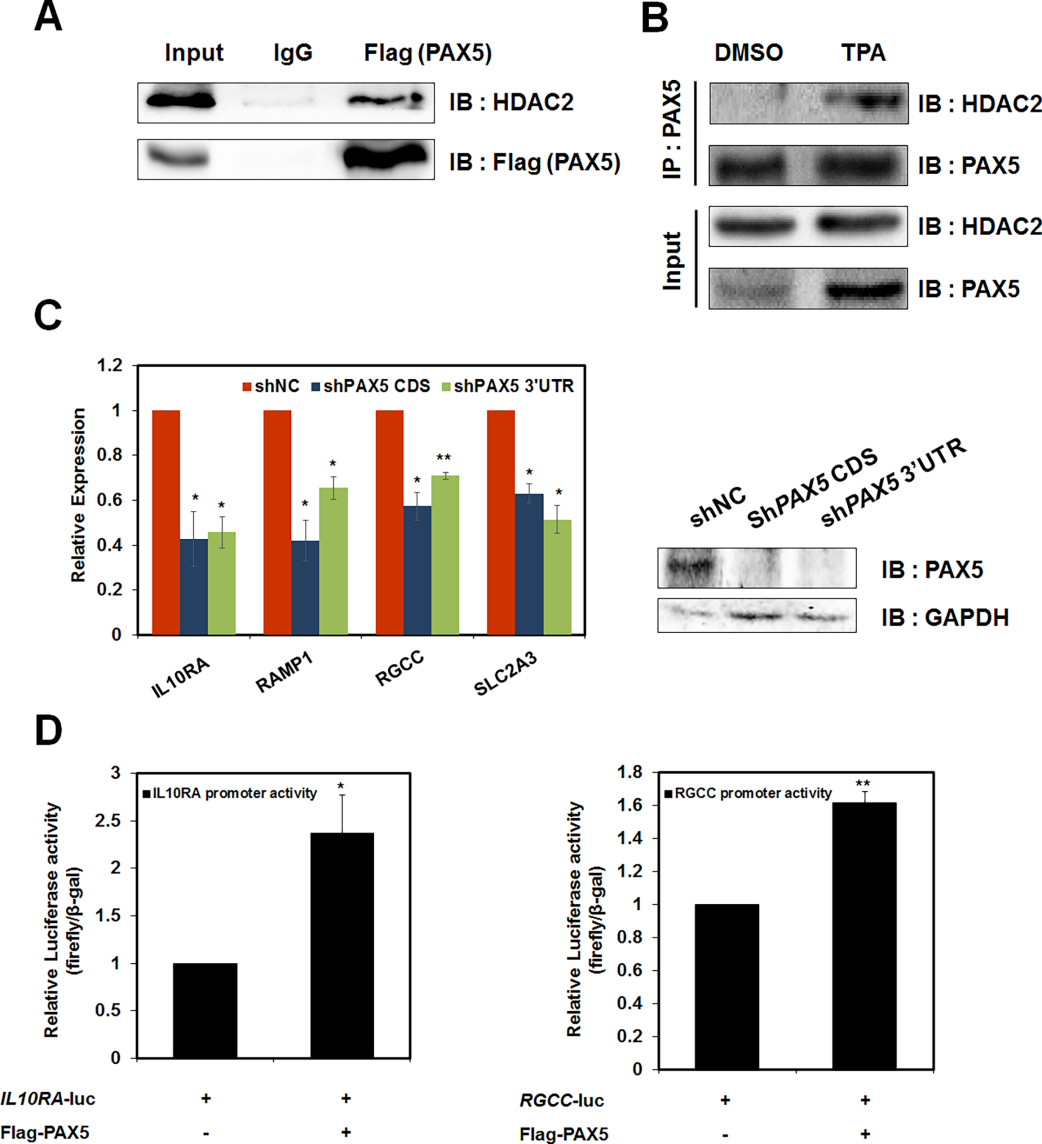
Recruitment of HDAC2 and PAX5 to the promoters of target genes. (A) Flag-tagged PAX5 was overexpressed in HEK293T cells. Extracts were immunoprecipitated with anti-Flag antibodies. Associated proteins were eluted, resolved by SDS-PAGE, and immunoblotted with anti-Flag and anti-HDAC2 antibodies. (B) Differential changes in interaction between HDAC2 and PAX5 48 hrs after treatment with 32 nM TPA were determined by IP. Extracts from TPA- or DMSO-treated HL-60 cells were immunoprecipitated with anti-PAX5 antibodies. HDAC2 that interacted with PAX5 in HL-60 cells was eluted and immunoblotted with a HDAC2 antibody. (C) The mRNA levels of atypically active genes in *PAX5*-depleted HL-60 cells were determined by qPCR. PAX5 protein level was detected by western blotting. These data were normalized by GAPDH. (D) HEK293T cells were co-transfected with pCMV-Flag-PAX5 and pGL3.0-*IL10RA* or pGL3.0-*RGCC* promoters. Luciferase activities were measured 48 hrs after transfection, and normalized to that of β-galactosidase. (C–D) All results represent at least three independent experiments (± SEMs). * *P* < 0.05, ** *P* < 0.01.

First, we checked PAX5 expression level in TPA-treated HL-60 cells. Consistent with previous data, the protein and mRNA levels of PAX5 increased (S3 Fig) [34]. To further determine whether the activity of HDAC2 was related to PAX5 functions on the promoters of atypically active genes, we examined the interactions between HDAC2 and PAX5. In a previous study, HDAC2 was reported to interact with PAX5 through Daxx [32]. In addition, PAX5 was shown to act as both a transcriptional activator and repressor in B cells via interactions with specific proteins such as Daxx, and the transcriptional activity modulated by Daxx was affected by interaction partners, including histone acetyltransferases or histone deacetylases [32, 33, 35]. In this study, we hypothesized that HDAC2 regulated target gene expression by recruiting PAX5 to the promoters of target genes independently of histone deacetylase activity. To test this hypothesis, we first overexpressed Flag-tagged PAX5 in HEK293T cells and observed the interaction between PAX5 and HDAC2 by IP (Fig 3A). During TPA-induced HL-60 cell differentiation, interactions between HDAC2 and PAX5 increased, suggesting the possibility that HDAC2 and PAX5 worked together for HL-60 cell differentiation (Fig 3B). We next analyzed the expression of atypically active genes in *PAX5* knockdown cells. The depletion of *PAX5* resulted in decreased gene expression in HL-60 cells, indicating that PAX5 functioned as a transcriptional activator of atypically active genes (Fig 3C). Subsequently, we performed a luciferase assay to measure the transcriptional activity of PAX5 at target gene promoters. Overexpression of PAX5 in HEK293T cells enhanced transcriptional activity of the *IL10RA* and *RGCC* promoters (Fig 3D). These data indicated that PAX5 increased the transcription of atypically active genes, and collectively these data suggested that HDAC2 and PAX5 interacted with each other to upregulate atypically active gene expression during the differentiation of TPA-induced HL-60 cells.

### HDAC2 recruits PAX5 to the promoters of atypically active genes

**Fig 4:**
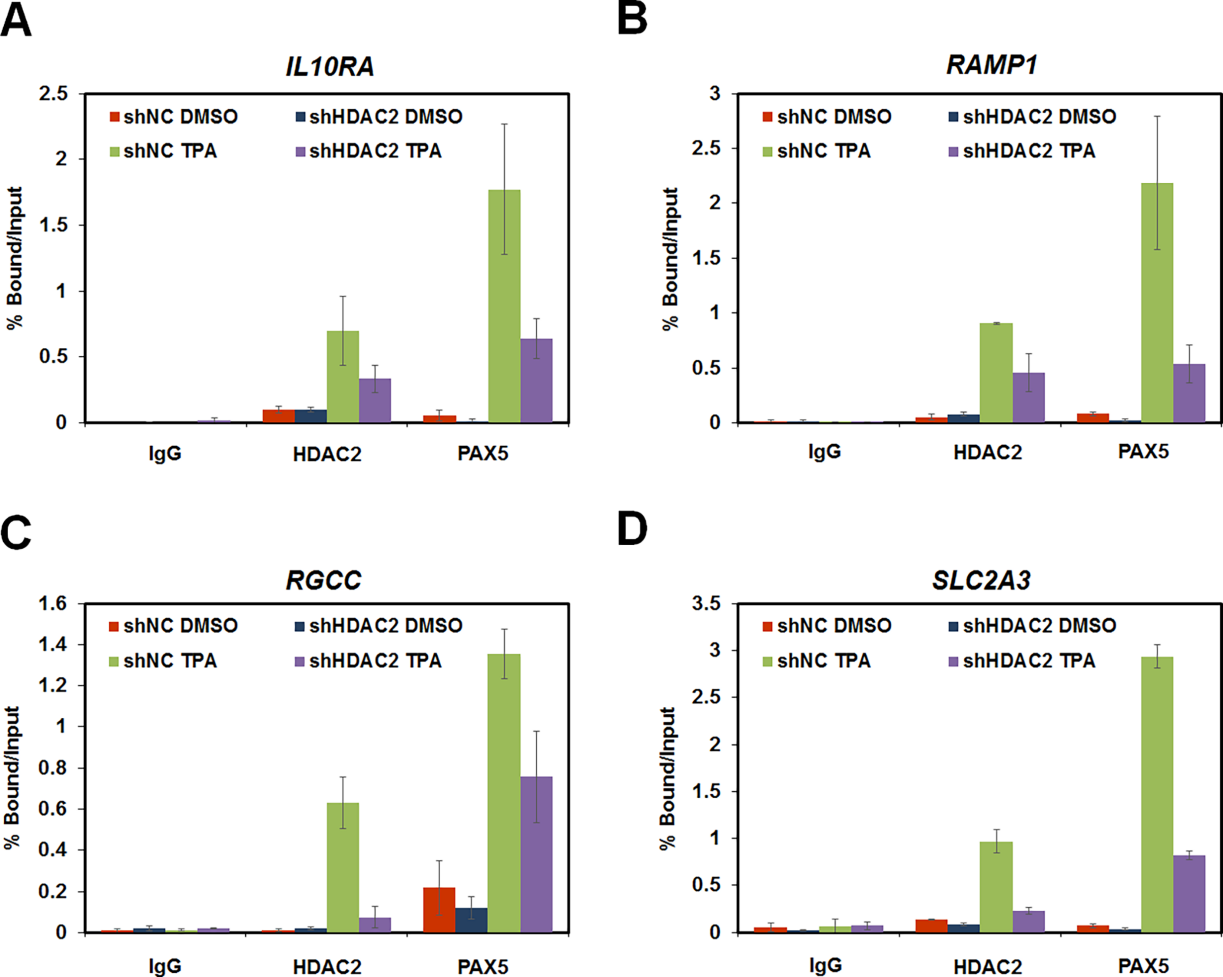
HDAC2 regulated atypically active gene expressions via recruitment of PAX5 to gene promoters. (A–D) ChIP analyses of the promoters of target genes in TPA-treated shNC and sh*HDAC2* 3′UTR HL-60 cells were examined using antibodies against HDAC2, PAX5, and mouse IgG by qPCR. These results are shown as means ± SDs (n = 3). (A) *IL10RA*, (B) *RAMP1*, (C) *RGCC*, and (D) *SLC2A3*.

Since we found that HDAC2 and AcH3 occupancies on the promoters of atypically active genes increased after TPA treatment of HL-60 cells, we performed ChIP-qPCR to determine whether PAX5 was recruited to the promoters of these genes during HL-60 cell differentiation by TPA treatment (Fig 4A–4D). As expected, TPA induced the recruitment of HDAC2 as well as PAX5 to the promoters of *IL10RA*, *RAMP1*, *RGCC*, and *SLC2A3*. In contrast, depletion of *HDAC2* impaired PAX5 recruitment (Fig 4A–4D). Collectively, these data indicate that the mechanism underlying gene activation by HDAC2 was enzyme activity independent and involved the recruitment of the transcriptional co-activator PAX5 to atypically active gene promoters during TPA-induced HL-60 cell differentiation.

### HDAC2 and PAX5 affect the HL-60 cell differentiation via co-regulation of atypically active genes

**Fig 5:**
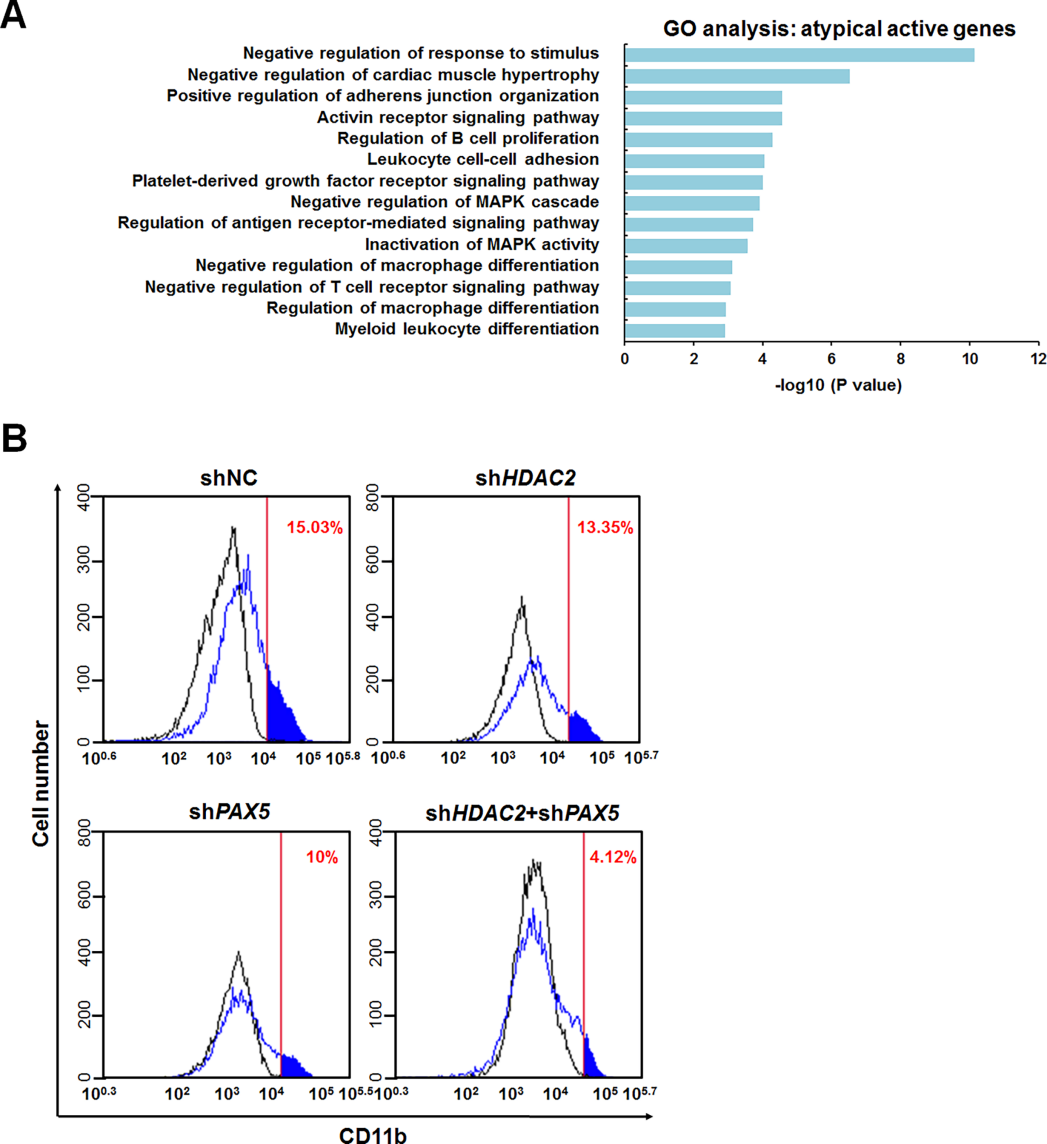
Knockdown of *HDAC2* in HL-60 cells caused differentiation inhibition when *PAX5* was also depleted. (A) GO terms of atypically active genes regulated by HDAC2 were analyzed in gene ontology consortium. X-axis represents the adjusted P-value transformed by -log10, and Y-axis denotes the enriched GO terms. (B) HL-60 cell differentiation was measured in control and sh*HDAC2* 3’UTR and/or sh*PAX5* 3’UTR HL-60 cells by CD11b staining. Cells were stained with CD11b-PE for 1 hr, and analyzed by FACS.

Gene ontology (GO) term analysis using gene ontology consortium (http://geneontology.org/page/go-enrichment-analysis) showed that the atypical genes were related to many cellular processes including leukocyte cell-cell adhesion, regulation of MAPK cascade, cellular response to stimulus and myeloid cell differentiation, indicating that enzyme activity-independent regulatory functions of HDAC2 are crucial for HL-60 cell differentiation (Fig 5A) [36, 37]. To investigate the functions of HDAC2 and PAX5 in the differentiation of HL-60 cells by TPA, we counted CD11b, HL-60 differentiation marker, expressing cells during differentiation by TPA in *HDAC2* and/or *PAX5* depleted HL-60 cells by FACS analysis (Fig 5B). Interestingly, knockdown of *PAX5* inhibited the differentiation of HL-60 cells while *HDAC2* depletion had little effects (Fig 5B). Our data showed that HDAC2 recruited PAX5 to atypically active genes and that regulated their expression levels, suggesting that HDAC2 and PAX5 might cooperate in HL-60 cell differentiation (Fig 4). Therefore, to test this possibility, we performed *PAX5/HDAC2* double knockdown experiments for analysis of differentiation of HL-60 cells. Although individual knockdown of *PAX5* or *HDAC2* has a limited effect on differentiation of HL-60 cells, the *PAX5/HDAC2* double knockdown showed a dramatic decrease in differentiation of HL-60 cells (Fig 5B). These results demonstrate that HDAC2 and PAX5 have individual and combined effects on the stimulation of differentiation of TPA-treated HL-60 cells.

## Discussion

In this study, we performed ChIP-seq profiling to determine the role of HDAC2 during HL-60 cell differentiation in response to TPA. Based on the ChIP-seq analysis data, we categorized the genes that were occupied by active markers, including AcH3 and RNA Pol II, into two major groups according to the recruitment of HDAC2 to promoters. Atypically active genes showed increased AcH3 levels with increased HDAC2 recruitment, indicating that HDAC2 might function in an enzyme activity-independent manner. Expression of the atypically active genes also increased after treatment with TPA and decreased after depletion of *HDAC2* in HL-60 cells. The enzyme-independent activity of HDAC2 included the recruitment of the transcriptional activator PAX5 to the target gene promoters. Interactions between HDAC2 and PAX5 were strengthened during HL-60 cell differentiation, and *HDAC2* depletion reduced PAX5 occupancies at the promoters, suggesting that HDAC2 is crucial for the appropriate recruitment of PAX5 to promoters of the genes. HDAC2 and PAX5 could affect HL-60 cell differentiation by TPA treatment, via the regulation of atypically active genes.

We performed ChIP-seq with antibodies against two histone H3 modifications, RNA Pol II, and HDAC2 to characterize the differentiation-specific chromatin changes in the HL-60 cells with TPA treatment. Interestingly, we found that HDAC2 was positively correlated with AcH3 and RNA Pol II peaks at atypically active genes, and contributed to transcriptional activation. We could suggest two potential hypotheses to explain a mechanism of HDAC2 to activate transcription of certain genes. First, HDAC2 may modulate the regulatory activity of transcription factors by removing an acetyl group. Previous studies have shown that the acetylation of transcriptional repressor BCL6 by p300 strongly enhanced its activity. The acetylated BCL6 has critical functions in different leukemia [38, 39]. It is possible that HDAC2 interacts and deacetylates lysine residue on BCL6, thereby resulting in repression of BCL6 transcriptional repressor activity. Second, we hypothesized that HDAC2 plays a positive role in the active transcription of genes by interacting with co-activators. Interestingly, we found the interaction between HDAC2 and PAX5 during HL-60 cell differentiation. Although the expression of PAX5 increased in TPA-treated HL-60 cells, PAX5 was recruited to the promoter regions of atypically active genes via HDAC2. In addition, HL-60 cell differentiation was inhibited in depletion of *PAX5*, but not that of HDAC2 in TPA-treated HL-60 cells. It might be caused by enzyme activity of HDAC2. Previous studies showed that HDAC inhibitors induce differentiation of leukemia by inhibiting the catalytic activities of HDAC2 [18, 40]. Although the individual *HDAC2* knockdown has little effect on HL-60 cell differentiation, we showed that double knockdown of HDAC2 and PAX5 severely inhibited HL-60 differentiation. It suggested that the enzyme activity-independent functions of HDAC2 via cooperating with PAX5 are also important for cell differentiation by regulating atypically active genes.

Valproic acid (VPA), a HDAC2 inhibitor, has been investigated in several clinical AML studies [18, 40]. In this case, lower concentration of VPA treatment accelerated differentiation of primary blasts from the bone marrow of AML patient while higher concentration showed little effects [40]. Furthermore, recent study showed that high concentration of VPA induced HDAC2 degradation [41]. HDAC2 degradation by high VPA level may lose not only enzyme activities, but also enzyme activity-independent roles, which facilitated leukemia differentiation in this study. Further studies will determine the detailed mechanisms of HDAC2 and whether the signaling pathways investigated in this study play another role of HDAC2, such as deacetylation of transcription factors during HL-60 cell differentiation.

Atypically active genes which were regulated by HDAC2 and PAX5 during HL-60 cell differentiation, participated in many cellular processes. Specifically, the IL10 receptor has functions in cytokine-cytokine receptor interaction and JAK-STAT signaling pathway, which are important for proliferation and survival of leukemia cells [8, 42]. Glucose transporter 3 (GLUT3) is encoded by the *SLC2A3* gene and facilitates glucose uptake across the plasma membrane. Glucose transporter proteins play a critical role at several cancer progression stages in various cancers and leukemia [43]. Furthermore, RGCC functions in the regulation and progression of cell cycle in different cells, implying a potential activity in HL-60 cell differentiation [44, 45]. Therefore, the study of enzyme activity-independent regulation by HDAC2 can be an attractive model for understanding the mechanisms of leukemia cell differentiation. As such, application of HDAC2 with TPA treatment for the leukemia therapy should be considered cautiously.

## Supporting information

S1 Fig ChIP-seq profiles of AcH3, RNA Pol II, HDAC2, and H3K27me3 of atypically activated genes in TPA-treated HL-60 cells. ChIP-seq tracks of AcH3, RNA Pol II, HDAC2, and H3K27me3 in HL-60 cells treated with DMSO or TPA along the *RGCC*, *BCL2A1*, *CDK8*, and *ABHD17B* loci.
(TIF)

S2 Fig HDAC2 was recruited to the promoters of atypically active genes and these genes were activated during HL-60 cell differentiation. (A-C) HL-60 cells were treated with TPA (32 nM) or DMSO for 48 hrs. (A) The *CD11b* mRNA level was measured by qPCR. (B) The recruitments of AcH3, H3K27me3, HDAC2, and RNA Pol II at the promoters of atypically active genes and typically active and repressed genes in TPA-treated HL-60 cells were analyzed. The data were normalized by input. These results are shown as means ± SDs (n = 3). (C) The differential genes expression changes during HL-60 cell differentiation were confirmed by qPCR. These data were normalized by *GAPDH*. All results represent at least three independent experiments (± SEMs). * *P* < 0.05, ** *P* < 0.01, *** *P* < 0.001.
(TIF)

S3 Fig The expression level of PAX5 increased during HL-60 cell differentiation by TPA treatment. (A) The protein level of PAX5 in HL-60 during differentiation was detected by western blotting. (B) The mRNA level of *PAX5* in TPA-treated HL-60 cells was determined by qPCR. These data were normalized by *GAPDH*. All results represent at least three independent experiments (± SEMs). * *P* < 0.05.
(TIF)

S1 Table Primers used in this study. (XLSX)
