# Supplementary figures and images for: Deacetylase activity-independent transcriptional activation by HDAC2 during TPA-induced HL-60 cell differentiation
Source: PLoS One. 2018 Aug 24;13(8):e0202935. doi: 10.1371/journal.pone.0202935 (PMC6108480; doi:10.1371/journal.pone.0202935)

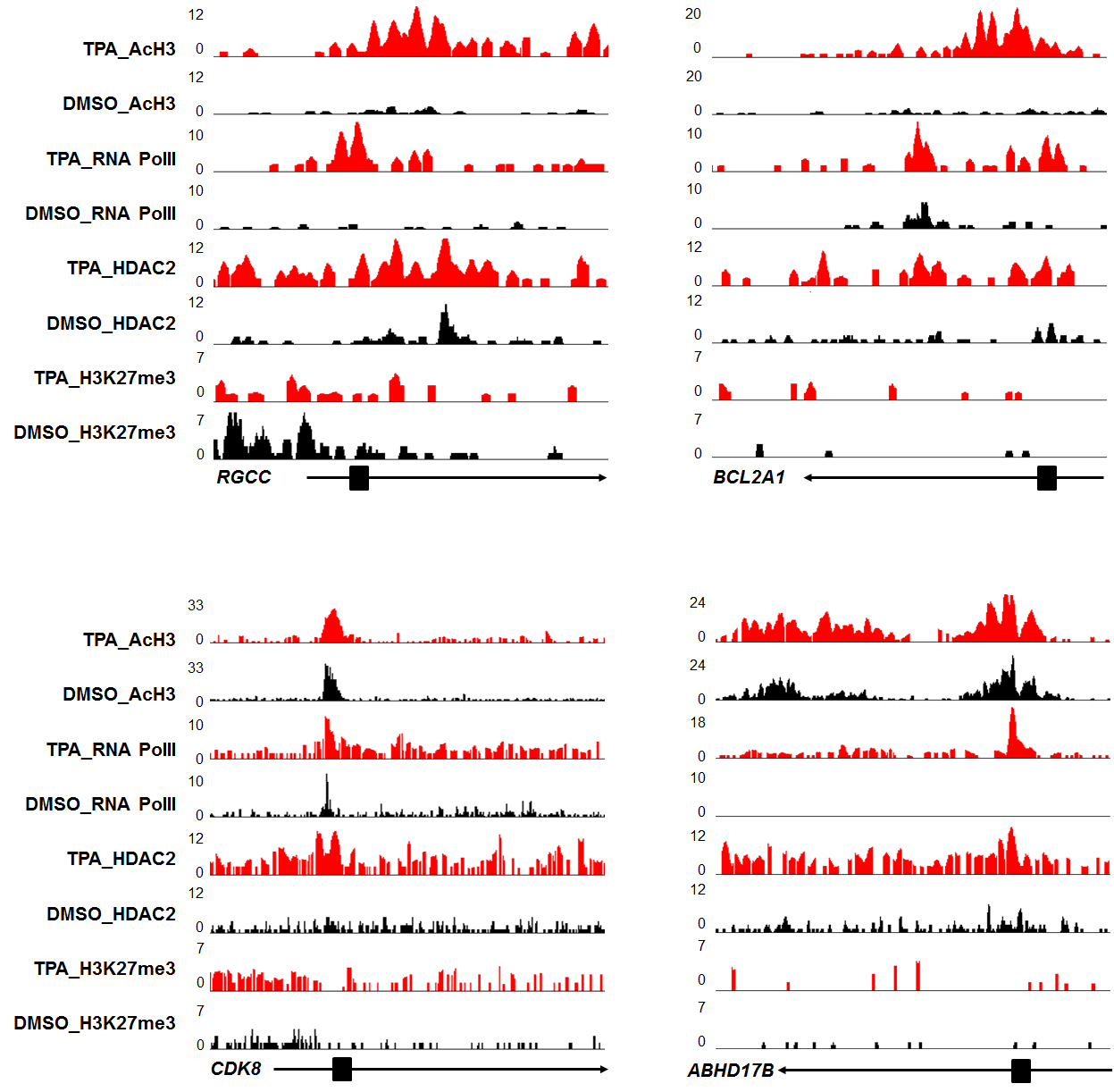

Supplement: S1 Fig — ChIP-seq tracks of AcH3, RNA Pol II, HDAC2, and H3K27me3 in HL-60 cells treated with DMSO or TPA along the RGCC, BCL2A1, CDK8, and ABHD17B loci. (TIF) [file pone.0202935.s001.tif]

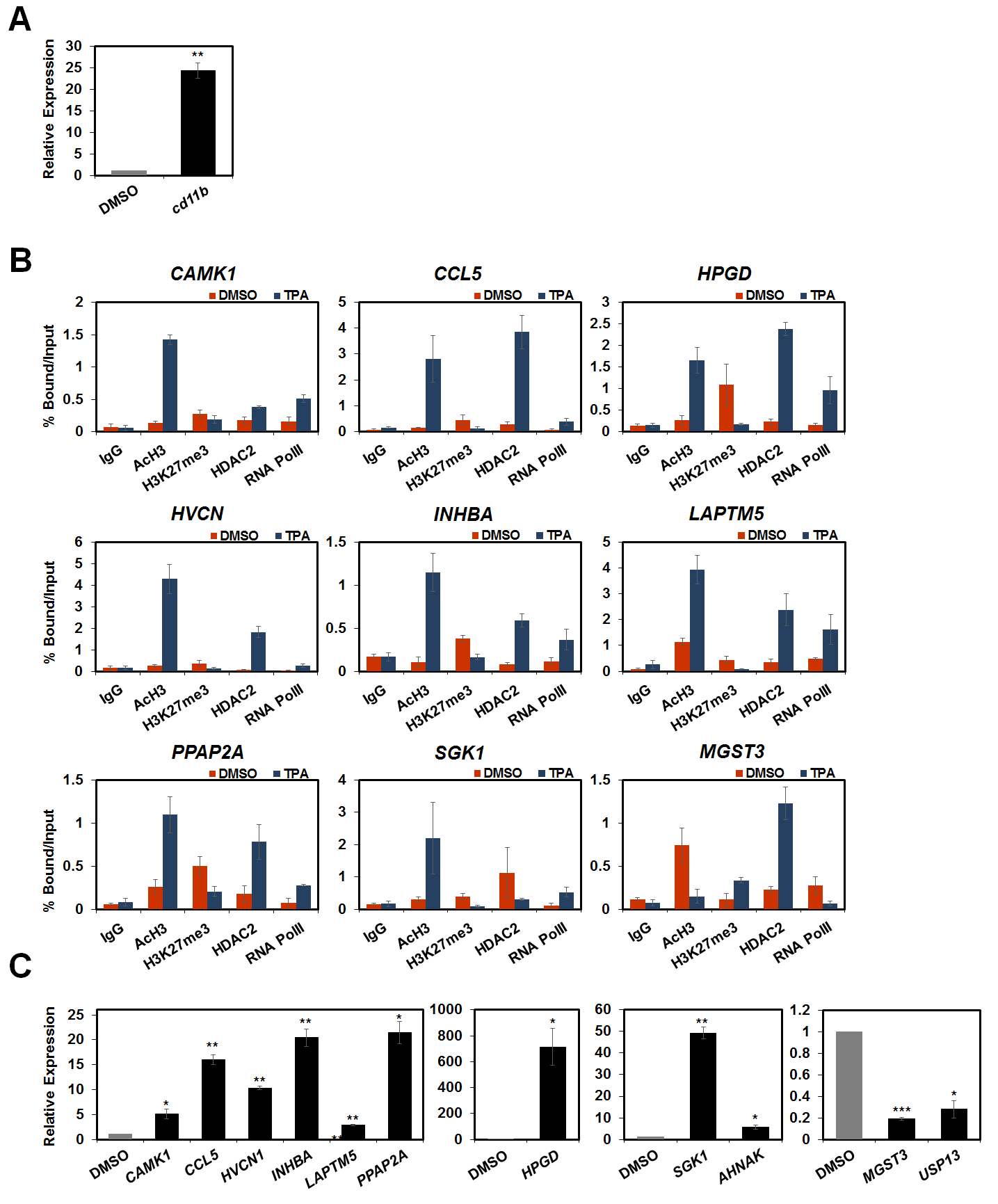

Supplement: S2 Fig — (A-C) HL-60 cells were treated with TPA (32 nM) or DMSO for 48 hrs. (A) The CD11b mRNA level was measured by qPCR. (B) The recruitments of AcH3, H3K27me3, HDAC2, and RNA Pol II at the promoters of atypically active genes and typically active and repressed genes in TPA-treated HL-60 cells were analyzed. The data were normalized by input. These results are shown as means ± SDs (n = 3). (C) The differential genes expression changes during HL-60 cell differentiation were confirmed by qPCR. These data were normalized by GAPDH. All results represent at least three independent experiments (± SEMs). * P < 0.05, ** P < 0.01, *** P < 0.001. (TIF) [file pone.0202935.s002.tif]

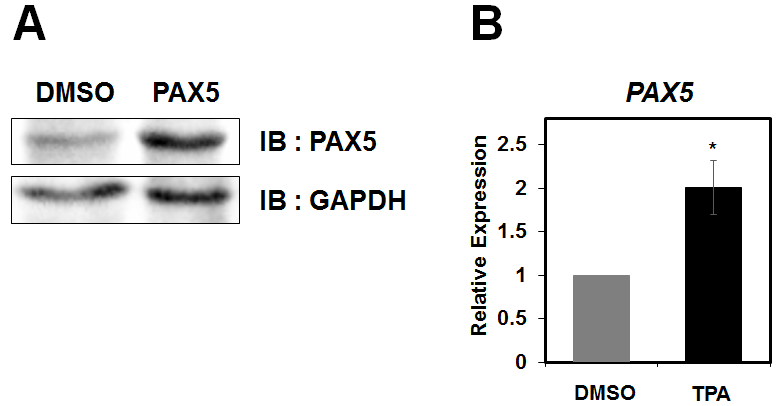

Supplement: S3 Fig — (A) The protein level of PAX5 in HL-60 during differentiation was detected by western blotting. (B) The mRNA level of PAX5 in TPA-treated HL-60 cells was determined by qPCR. These data were normalized by GAPDH. All results represent at least three independent experiments (± SEMs). * P < 0.05. (TIF) [file pone.0202935.s003.tif]
